# Determinants of the Public’s Behavioral Intention to Adopt AI-Assisted Lung Cancer Screening: An Extended UTAUT Model Integrating Trust and Risk

**DOI:** 10.3390/healthcare14070945

**Published:** 2026-04-03

**Authors:** Langwei Yan, Xue Bai, Xiurong Lin, Jingfu Lai, Shuhan Sun, Hengwei Chen, Ruqing Liu, Ruwei Hu

**Affiliations:** 1 School of Public Health, Sun Yat-Sen University, Guangzhou 510080, China; yanlw7@mail2.sysu.edu.cn (L.Y.); baix23@mail2.sysu.edu.cn (X.B.); linxr57@mail2.sysu.edu.cn (X.L.); laijf8@mail2.sysu.edu.cn (J.L.); sunshh25@mail2.sysu.edu.cn (S.S.); chenhw68@mail2.sysu.edu.cn (H.C.); 2 School of Law, Sun Yat-Sen University, Guangzhou 510080, China

**Keywords:** AI-assisted screening, lung cancer, determinants, UTAUT, perceived risk, doctor–patient trust

## Abstract

**Background:** The integration of artificial intelligence (AI) into lung cancer screening offers significant potential; however, public adoption of AI-assisted lung cancer screening remains inconsistent and poorly understood. A robust understanding of the psychological and social determinants underlying adoption is critical to inform evidence-based implementation strategies. **Objective:** This study aims to identify the key factors that influence the public’s Behavioral Intention (BI) to adopt AI-assisted lung cancer screening. We built on the Unified Theory of Acceptance and Use of Technology (UTAUT) and integrated Doctor–Patient Trust and Perceived Risk into the framework to examine the associations between these medically specific factors, together with traditional adoption variables, and the public’s BI. **Methods:** A cross-sectional survey was conducted among 971 residents in China from September to November 2025. Based on the extended UTAUT, a measurement instrument was developed and refined through expert consultations and pilot testing. Exploratory factor analysis (EFA) was performed to validate the questionnaire’s construct validity. Hypothesis testing was then carried out via Partial Least Squares Structural Equation Modeling (PLS-SEM) to evaluate the measurement model and examine the structural relationships among latent constructs. **Results:** EFA results indicated a seven-factor structure (KMO = 0.897, *p* < 0.001). The structural model accounted for 35.0% of the variance in BI. Social Influence (β = 0.292, *p* < 0.001), Facilitating Conditions (β = 0.156, *p* < 0.001), Performance Expectancy (β = 0.101, *p* = 0.004), Doctor–Patient Trust (β = 0.107, *p* = 0.002) were positively associated with BI, while Perceived Risk (β = −0.106, *p* < 0.001) showed a negative association. Furthermore, Doctor–Patient Trust was significantly and negatively associated with Perceived Risk (β = −0.168, *p* < 0.001), suggesting a potential mediating pathway from trust to intention (Indirect Effect = 0.018, *p* = 0.003). **Conclusions:** Adoption of AI-assisted lung cancer screening appears to be associated not only with perceived utility but also with trust in medical professionals and Perceived Risk. These findings suggest the importance of integrating technological innovation with strategic public education and tailored communication strategies to foster its adoption. Public health interventions should leverage physician endorsements and promote AI awareness to support informed, trust-based engagement with AI technologies.

## 1. Introduction

Early detection through screening remains one of the most effective strategies for reducing lung cancer mortality. In recent years, AI-assisted lung cancer screening has emerged as a promising approach to enhance early detection. Evidence indicates that these systems have been shown to identify pulmonary nodules with diagnostic accuracy that is comparable to or even exceeds that of expert radiologists, while significantly reducing false-positive rates and diagnostic time [1,2]. However, successful clinical implementation depends not only on technical performance but also on individuals’ willingness to accept and use these technologies [3,4]. Understanding the determinants of the public’s Behavioral Intention (BI) to adopt AI-assisted lung cancer screening is therefore critical for translating technological innovation into population health impact.

Research on AI health technology acceptance has increasingly recognized that adoption decisions are shaped by a complex interplay of cognitive evaluations, affective responses, social influences, and contextual facilitators. Aras et al. demonstrated that public acceptance of AI in healthcare is influenced by the interplay between demographic, socioeconomic (e.g., healthcare access), and cultural factors (e.g., trust) [5]. Dingel and colleagues’ meta-analysis of AI clinical decision support systems confirmed that performance expectancy consistently emerges as the strongest predictor of acceptance intentions, yet identified trust as the most influential factor when included as an extension construct [6]. These findings underscore that BI is determined not merely by what individuals think about the technology, but by how they feel about it, who influences them, and what resources enable their engagement.

However, existing research on AI health technology acceptance exhibits limitations when applied to AI-assisted lung cancer screening specifically. The initial focus has largely been on healthcare professionals rather than the general public. Swathi and colleagues examined the BI of healthcare professionals regarding AI adoption in healthcare service delivery [7], while Schaffernak and colleagues investigated sociotechnical influences on ophthalmologists’ adoption of AI-assisted clinical decision support systems [8]. These professional-centered findings may not transfer to public acceptance, as screening decisions involve different psychological dynamics. Subsequent research examining general public AI acceptance have focused on consumer health applications or diagnostic tools rather than preventive screening. Su and colleagues investigated factors influencing users’ intentions to adopt AI health assistants [9], while Lee and colleagues extended UTAUT to examine ChatGPT adoption among general users [10]. These contexts differ fundamentally from cancer screening, where the absence of symptoms, the fear of diagnosis, and the probabilistic nature of screening results create unique psychological barriers. Most critically, no empirical study to date has specifically investigated the public’s BI to adopt AI-assisted lung cancer screening.

The determinants of acceptance for AI-assisted lung cancer screening are likely distinct from those for general AI health applications or traditional non-AI screening. Multifaceted barriers exist: At the individual level, screening participants face fear of cancer diagnosis, concerns about radiation exposure from low-dose CT, and insufficient awareness of screening benefits; at the interpersonal level, the quality of doctor–patient relationships, strength of physician recommendations, and shared decision-making processes significantly influence participation willingness; at the organizational level, insurance coverage, appointment accessibility, and care coordination constitute critical barriers. Moreover, the introduction of AI technology adds novel psychosocial dimensions. As highlighted by Mascalchi and colleagues, explainability constitutes a critical determinant of AI-assisted lung cancer screening adoption, extending beyond mere technical performance. The limited explainability of AI systems, however, may constrain their clinical utility and wider adoption [11]. This context presents distinctive challenges: screening often involves probabilistic findings such as indeterminate pulmonary nodules and potentially serious consequences associated with AI misdiagnosis [12,13], heightening individuals’ sensitivity to perceived risk. As patients typically depend on physician guidance when confronting novel medical technologies involving uncertain outcomes [14,15], Doctor–Patient Trust (DPT) becomes crucial. Thus, Perceived Risk (PR) and DPT may be important factors associated with public acceptance of AI-assisted screening, yet these dynamics remain insufficiently explored.

To adequately capture these multidimensional determinants, this study integrates the Unified Theory of Acceptance and Use of Technology (UTAUT) with medical context-specific constructs. The UTAUT framework, developed by Venkatesh and colleagues, integrates eight previous theories including the Technology Acceptance Model (TAM) and the Theory of Planned Behavior (TPB), identifying Performance Expectancy (PE), Effort Expectancy (EE), Social Influence (SI), and Facilitating Conditions (FCs) as the core predictors of BI [16]. As one of the most widely adopted theoretical frameworks in technology acceptance research, the UTAUT has been successfully extended in healthcare studies by incorporating factors such as perceived security [17,18], perceived threat and trust [19], and anxiety and data security [20]. These extensions demonstrate that augmenting UTAUT with domain-specific constructs strengthens its explanatory capacity.

This study therefore aims to identify the key determinants of public BI to adopt AI-assisted lung cancer screening. By developing an extended model that incorporates PR and DPT into the UTAUT framework, we seek to explore the underlying mechanisms associated with the public’s willingness to adopt AI-assisted screening. This integrated approach allows for a more nuanced understanding of the ways in which technical, social, and relational factors are associated with screening decisions, providing evidence-based insights for the clinical implementation of medical AI. As the first empirical investigation of this specific phenomenon, this study establishes foundational evidence for theoretical development and practical advancement in this emerging field.

## 2. Research Model and Hypotheses

### 2.1. Behavioral Intention to Adopt AI-Assisted Lung Cancer Screening

BI is shaped by individual attitudes and subjective norms and serves as a predictor of actual behavior [16,21]. Grounded in the Theory of Reasoned Action (TRA) and the TPB, BI captures the motivational factors associated with an individual’s decision to adopt a specific technology [22,23]. The TPB posits that BI is shaped by three determinants: attitude toward the behavior, subjective norms, and perceived behavioral control [23,24]. This tripartite structure has demonstrated robust predictive validity across diverse domains, from health behaviors to technology adoption [25]. In digital health, BI goes beyond simply agreeing to use a device; it reflects a user’s innovation readiness to rely on new tools for their health [26]. This conceptualization is particularly relevant for AI-assisted healthcare technologies, where balancing AI’s perceived benefits and potential risks is essential [21]. In the specific context of AI-assisted lung cancer screening, BI captures the motivational readiness to undergo screening that incorporates AI-based image interpretation. BI in healthcare technology adoption may manifest through multiple behavioral orientations rather than a single behavioral expression. In addition to an individual’s willingness to personally use a technology, individuals may also evaluate whether the technology represents an improvement over existing clinical practices and whether they would recommend it to others [27]. These related expressions reflect different manifestations of an underlying psychological readiness to accept the integration of new technologies into healthcare services. In the present study, these dimensions were modeled as indicators of a single latent construct because they collectively capture an individual’s overall acceptance orientation toward AI-assisted screening. This operationalization is consistent with prior UTAUT-based research in digital health, where BI reflects a general motivational readiness to adopt a technology rather than a narrowly defined behavioral act [27,28,29].

### 2.2. The Unified Theory of Acceptance and Use of Technology

The UTAUT was initially proposed by Venkatesh et al. [16] in 2003 as a comprehensive framework to explain technology adoption, drawing upon the synthesis of eight prominent models, including the TRA, the Technology Acceptance Model (TAM), and the Theory of Planned Behavior. The basic UTAUT framework assumes that an individual’s BI to use a system is determined by four core constructs: PE, EE, SI, and FC. According to Venkatesh et al., PE is the degree to which an individual believes that using a system will help them attain gains, while EE represents the degree of ease associated with system use. SI is defined as the extent to which an individual perceives that important others believe they should use the new system, and FCs refer to the perception of whether organizational and technical infrastructure exists to support the system.

### 2.3. Hypotheses Development

Building upon the original UTAUT framework, this study examines the determinants of public acceptance of AI-assisted lung cancer screening. While the UTAUT provides a well-established explanation for technology adoption through four core predictors—PE, EE, SI, and FC—the healthcare context introduces additional considerations that extend beyond general technology use. In particular, decisions related to medical screening involve strong elements of clinical trust and perceived health risk. Therefore, in addition to the four core UTAUT constructs, this study incorporates DPT and PR to capture the psychological and clinical mechanisms that may shape individuals’ acceptance of AI-assisted screening technologies. The resulting framework retains the core structure of UTAUT while extending it to better reflect the decision-making dynamics present in healthcare settings.

#### 2.3.1. Core UTAUT Predictors of Behavioral Intention

The UTAUT and its extensions have been widely used to explain people’s behaviors in healthcare technology acceptance. For instance, the UTAUT has been successfully applied to analyze the adoption of health informatics [30], medical imaging [31], and mHealth Applications [32]. In the context of radiology, UTAUT has proven effective in predicting patients’ willingness to utilize automated image interpretation systems [33].

In this research, we adapt the original UTAUT framework to the context of AI-assisted lung cancer screening. It is important to note that this research focuses on BI rather than actual usage behavior. Given that AI-assisted lung cancer screening is not yet routinely available for the general public, individuals may lack direct experience with such systems.

In this context, PE transcends mere efficiency; it embodies the patient’s perception of the technology as a diagnostic safety net. Given that early detection is the most critical modifiable factor for improving lung cancer survival, individuals are more likely to form positive intentions if they believe AI augments the sensitivity and accuracy of nodule detection beyond conventional radiology, thereby offering a tangible survival benefit. Thus, we hypothesize the following:

**H1.**  *Performance Expectancy (PE) has a positive influence on the Behavioral Intention (BI) to use AI-assisted lung cancer screening.*

For AI-assisted screening, EE is not primarily about operating an interface, but rather the cognitive and procedural burden of integrating the technology into the screening pathway. This includes the clarity of pre-screening instructions, the seamless integration of AI analysis into the standard low-dose CT scan procedure, and the accessibility and comprehensibility of AI-generated results, whether delivered via patient portals or explained by clinicians. A streamlined, low-effort process reduces barriers to initiation. Therefore,

**H2.**  *Effort expectancy (EE) has a positive influence on the Behavioral Intention (BI) to use AI-assisted lung cancer screening.*

In healthcare, a recommendation from a trusted family member can be a powerful driver. Additionally, narratives from peers (e.g., lung cancer survivors in support groups) and the broader societal discourse around AI in medicine, as shaped by media and public health campaigns, significantly shape normative perceptions and intentions. Hence,

**H3.**  *Social Influence (SI) has a positive influence on the Behavioral Intention (BI) to use AI-assisted lung cancer screening.*

For AI-assisted screening, FCs encompass tangible, systemic enablers. These include the geographic and financial accessibility of screening centers offering AI technology, and the availability of insurance coverage or subsidies for the AI-augmented scan. Unlike EE, which is subjective, FCs reflect objective environmental support. We propose the following:

**H4.**  *Facilitating Conditions (FCs) have a positive influence on the Behavioral Intention (BI) to use AI-assisted lung cancer screening.*

#### 2.3.2. Trust and Risk in AI-Assisted Medical Decision-Making

In addition to the core UTAUT predictors, our study incorporates DPT and PR to address psychological safety and clinical uncertainty. PR involves the fear of potential misdiagnosis (performance risk) and concerns regarding sensitive biological data leakage (privacy risk) [34,35]. According to Prospect Theory, individuals are loss-averse, and high levels of PR can act as a significant barrier to the adoption of life-saving technologies [36]. Specifically, patients may be concerned about potential inaccuracies in AI diagnoses, unclear medical responsibility in case of error, or the possibility of unauthorized leakage of health information [37]. These concerns can increase their reluctance to trust AI technology. These PRs create a psychological burden that diminishes the willingness to use AI for health-related decisions.

On the other hand, DPT represents the patient’s positive expectation regarding the physician’s competence, benevolence, and integrity [38]. According to Trust Transfer Theory [39], trust can be transferred from a trusted intermediary (the physician) to a related technological entity (the AI system). When patients trust their physicians, they are more likely to extend this trust to AI technologies recommended or supervised by them, thereby bridging the technological divide. For instance, patients who believe that their doctors are capable of evaluating the accuracy and clinical validity of AI-generated results are more inclined to accept AI-assisted lung cancer screening. Similarly, when physicians are perceived as willing to explain AI outputs and engage with patients’ concerns, confidence in both the technology and its clinical integration is strengthened. Therefore, we set the following hypotheses:

**H5.**  *Doctor–Patient Trust (DPT) has a positive influence on the Behavioral Intention (BI) to use AI-assisted lung cancer screening.*

**H6.**  *Perceived Risk (PR) has a negative influence on the Behavioral Intention (BI) to use AI-assisted lung cancer screening.*

Beyond its direct effect on BI, trust is also crucially associated with reduced PR. According to Luhmann’s sociological perspective on trust [40], trust is a factor associated with reduced complexity, allowing individuals to act despite incomplete information. In healthcare, trusted physicians are often viewed as professional “gatekeepers” who oversee and validate technological systems, which in turn is associated with reduced patient concerns about algorithmic errors, unclear accountability, and data misuse. Some studies in e-health and medical technology adoption have consistently shown that higher levels of trust are associated with lower levels of PR and with adoption intention [41,42].

Based on this reasoning, DPT is expected to be directly associated with BI and indirectly associated with adoption through PR. Therefore, we propose the following hypotheses concerning the mediating role of PR:

**H7.**  *Doctor–Patient Trust (DPT) has a negative influence on Perceived Risk (PR).*

**H8.**  *Perceived Risk (PR) mediates the relationship between Doctor–Patient Trust (DPT) and the Behavioral Intention (BI) to use AI-assisted lung cancer screening.*

**Figure 1:**
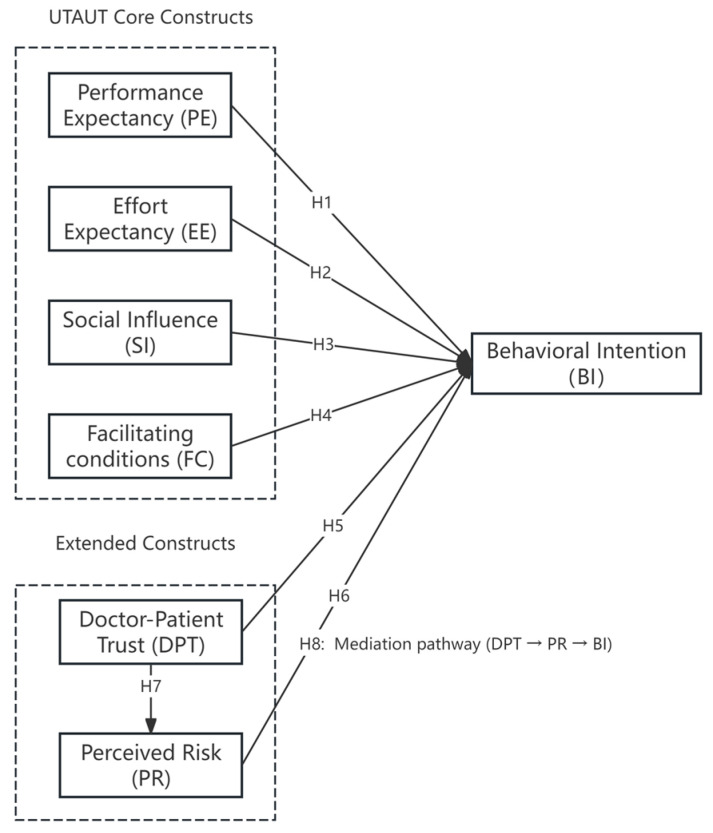
Proposed research model integrating UTAUT constructs with healthcare-specific factors influencing BI toward AI-assisted lung cancer screening. Note: The model integrates the four core constructs of the Unified Theory of Acceptance and Use of Technology (UTAUT)—Performance Expectancy (PE), Effort Expectancy (EE), Social Influence (SI), and Facilitating Conditions (FCs)—with two healthcare-specific constructs: Doctor–Patient Trust (DPT) and Perceived Risk (PR). The model hypothesizes that PE, EE, SI, FCs, DPT, and PR influence Behavioral Intention (BI). In addition, Doctor–Patient Trust is expected to reduce Perceived Risk (H7), and Perceived Risk mediates the relationship between Doctor–Patient Trust and Behavioral Intention (H8).

Understanding these determinants and their influence on public adoption can help policymakers and medical institutions pinpoint barriers to AI integration, ultimately enhancing the efficiency of lung cancer screening programs. The proposed research model along with its hypotheses is illustrated in Figure 1.

## 3. Materials and Methods

### 3.1. Measurement Instrument Development

The study instrument was a structured questionnaire developed based on the extended UTAUT framework. Measurement items were adapted from previously validated scales in the UTAUT and healthcare technology acceptance literature and were contextualized to the scenario of AI-assisted lung cancer screening (see Table 1).

The questionnaire consisted of two sections: participant demographics and the measurement of seven latent constructs (PE, EE, SI, FCs, PR, DPT, and BI). Content validity was established through a two-round Delphi process with multidisciplinary experts in public health, AI, and medical informatics, which resulted in a refined 22-item scale with strong consensus (Kendall’s W = 0.62) and high content validity (S-CVI = 0.87). Items with an I-CVI below 0.78 were removed.

Face validity was confirmed through interviews with five residents from diverse backgrounds to ensure the clarity, readability, and comprehensibility of the questionnaire items. A pilot study (n = 56) conducted between June and July 2025 demonstrated excellent internal consistency (Cronbach’s α = 0.935), with an average completion time of 10–15 min. All items used a five-point Likert scale (1 = strongly disagree, 5 = strongly agree). The positively framed item (PR1) measuring PR was reverse-worded (risk denial statements) to reduce acquiescence bias. The item was reverse-coded prior to statistical analysis so that higher scores across all items consistently reflected higher PR. Dimension scores were calculated as the mean of corresponding items. Based on their mean scores on the three BI items, participants were categorized into high BI (mean ≥ 4) and low BI (mean < 4) groups for subsequent comparative analyses.

### 3.2. Participants

This study employed a nationwide cross-sectional survey conducted from September to November 2025. The target population consisted of adults aged 18–65 years across all 31 provincial-level administrative regions in mainland China. A multi-stage stratified sampling strategy was used to obtain a geographically diverse sample. In the first stage, prefecture-level regions within each province were grouped according to population size, from which groups a subset of regions was randomly selected. In the second stage, county-level units within the selected prefectures were further sampled. Sampling quotas were established based on statistical yearbooks to broadly reflect the age and gender composition of the adult population.

Data were collected using standardized Computer-Assisted Telephone Interviewing (CATI) between September and November 2025. Participants were contacted via telephone using a CATI system. Prior to the survey, participants provided informed consent for their data to be collected and analyzed anonymously. The data collection procedure complied with the ISO 20252 standard [47], and all stored and analyzed data were fully anonymized. Trained interviewers conducted telephone interviews using the system to administer the structured questionnaire. Individuals with cognitive or language impairments that could compromise response validity were excluded from participation. The initial data collection yielded 1452 responses.

A total of 1452 interviews were completed during the fieldwork period. Following data collection, the dataset was screened using predefined data-quality procedures to ensure response completeness and reliability. Interviews in which respondents did not complete a sufficient number of items required for the measurement model were excluded from the analysis. Unreliable responses were also removed based on two criteria: highly repetitive answering patterns (defined as >50% consecutive identical responses) [48] and logical inconsistencies in reverse-coded items within a construct.

After screening, 971 valid responses were retained, yielding a final validity rate of 66.7%. The final sample size met the minimum requirement for comparing two groups (*n* = 774) [49].

This study protocol was approved by the Human Studies Committee of Sun Yat-Sen University on May 17, 2024 (No. IRB2024.100), following the Declaration of Helsinki-Ethical Principles for Medical Research Involving Human Subjects. All participants were briefed on the study objectives prior to participation and provided informed consent.

### 3.3. Methodology Analysis

The normality of continuous variables was assessed using the Shapiro–Wilk test. Variables with approximately normal distribution are presented as mean ± standard deviation (SD), whereas non-normally distributed variables are reported as median with first and third quartiles (median [Q1, Q3]). Categorical variables are summarized as frequencies and percentages, n (%). Group differences in categorical variables were examined using the Chi-square test, while differences in continuous construct scores (PE, EE, SI, FCs, DPT, and PR) between groups were compared using the Mann–Whitney U test.

To verify the validity of the questionnaire design, this study conducted an exploratory factor analysis (EFA). Prior to factor extraction, we evaluated the suitability of the data for factor analysis using the Kaiser–Meyer–Olkin (KMO) measure of sampling adequacy and Bartlett’s test of sphericity [50,51]. Principal component analysis (PCA) was employed as the extraction method, and factor rotation was performed to enhance interpretability. Item retention was determined based on established criteria: items with communalities above 0.40 and factor loadings exceeding 0.70 were retained for further analysis [52,53].

To assess the measurement model and structural model of the extended UTAUT framework, Partial Least Squares Structural Equation Modeling (PLS-SEM) was performed. This variance-based SEM technique is preferred over covariance-based SEM because it handles non-normal data distributions and analyzes both reflective and formative measurement models [54,55]. In addition, PLS-SEM has been widely adopted in information systems and healthcare technology acceptance research [56,57,58]. The assessment of the measurement model involved evaluating internal consistency reliability (Cronbach’s alpha and composite reliability), convergent validity (average variance extracted, AVE), and discriminant validity (Fornell–Larcker criterion and heterotrait–monotrait ratio, HTMT). Following the evaluation of the measurement model, the bootstrapping procedure (with 5000 resamples) was employed to test the significance of the path coefficients. The structural model was assessed by examining the path coefficients (β-values), standard deviations (STD), t-values, and *p*-values, and R-squared values. The t-statistic was used to determine significance. Paths with t-values above 1.96 were considered statistically significant at the 5% significance level [59].

To further examine whether the structural relationships in the proposed model remain stable across different demographic groups, additional stratified analyses were conducted using partial least squares multi-group analysis (MGA) [60]. Respondents were divided into subgroups based on educational attainment and age. Educational level was categorized as lower education (high school or below) and higher education (associate degree or above), while age was dichotomized into younger (<50 years) and older (≥50 years) groups. A permutation test was conducted to assess whether the structural path coefficients differed significantly between groups. The analysis estimated path coefficients for each subgroup and evaluated the statistical significance of the differences between groups based on 5000 permutations. A *p*-value below 0.05 was considered evidence of significant group differences.

Preliminary data checks, descriptive statistics, and EFA were conducted using IBM SPSS 24.0. The evaluation of the measurement model, structural model, and the MGA were performed using PLS-SEM implemented in SmartPLS 4.1. A two-tailed *p*-value < 0.05 was considered statistically significant.

## 4. Results

### 4.1. Characteristics of Participants

The demographic characteristics of the final sample (N = 971) are summarized in Table 2. The participants had a mean age of 37.2 years, with a nearly balanced gender distribution (50.4% male). The majority were urban residents (87.8%) with a bachelor’s degree or above (36.1%), and 61.5% reported prior awareness of medical AI. Comparisons between the high (n = 504, 51.8%) and low (n = 467, 48.2%) BI groups revealed statistically significant differences in several key variables. Specifically, the high BI group demonstrated a significantly higher level of prior awareness of medical AI (*p* < 0.001) and reported significantly higher median scores for PE, EE, SI, FCs, and DPT, along with significantly lower PR (all *p* < 0.001). In contrast, no significant differences were observed between the two groups in terms of sex, age, education level, household income, or place of residence (all *p* > 0.05).

### 4.2. Exploratory Factor Analysis

The KMO value was 0.897, and Bartlett’s test of sphericity was significant (χ^2^ = 6776.04, *p* < 0.001), confirming the data’s suitability for factor analysis. Most items demonstrated adequate communalities exceeding 0.40, suggesting that they contributed meaningfully to the extracted factors. However, SI3 was removed from further analysis due to a low factor loading (0.452) and cross-loadings across multiple factors. After removing SI3, the retained construct primarily reflects interpersonal normative influence, and the remaining items were subjected to Varimax rotation. All retained items exhibited strong factor loadings exceeding 0.70 on their respective factors (see Table 3). The rotated factor structure was consistent with the theoretical dimensions of the scale, indicating a clear and interpretable factor solution. Overall, the results demonstrate good construct validity, supporting the suitability of the questionnaire for subsequent analyses.

### 4.3. Measurement Model Evaluation

The reliability and validity assessment (Table 4) confirms that the measurement model is robust across all constructs. Outer loadings were greater than 0.708, demonstrating strong explanatory power for each latent variable [61]. The majority of item loadings exceeded this threshold, ranging from 0.709 to 0.865. Although two items (PR4 and BI2) exhibited loadings slightly below 0.7, they were retained because the AVE and CR values of their respective constructs remained above the recommended thresholds [61]. Although Cronbach’s alpha values ranged from 0.636 (BI) to 0.783 (SI), composite reliability (CR) was prioritized as the primary indicator of internal consistency, given its suitability for PLS-SEM. CR values were above 0.7, indicating good internal consistency [62].

According to Fornell and Larcker [63], AVE measures the amount of variance a construct captures from its indicators relative to the amount due to measurement error. An AVE value of 0.50 or higher is considered acceptable. In our study, the AVE values ranged from 0.580 to 0.669, confirming satisfactory convergent validity.

To ensure construct distinctiveness, discriminant validity was assessed using both the Fornell–Larcker criterion and the heterotrait–monotrait ratio (HTMT). As shown in Table 5, the bolded diagonal elements represent the square root of the AVE for each construct, which exceeded the correlations with all other constructs, satisfying the Fornell–Larcker criterion [63]. Furthermore, all HTMT values were below the recommended threshold of 0.85 [64,65], confirming that the constructs are empirically distinct.

### 4.4. Structural Model Assessment of Determinants

Collinearity assessment was performed to examine potential multicollinearity issues among predictor variables. The variance inflation factor (VIF) for all predictor variables ranged from 1.136 to 1.888, well below the commonly accepted threshold of five, indicating that multicollinearity is not an issue in this study [54].

**Figure 2:**
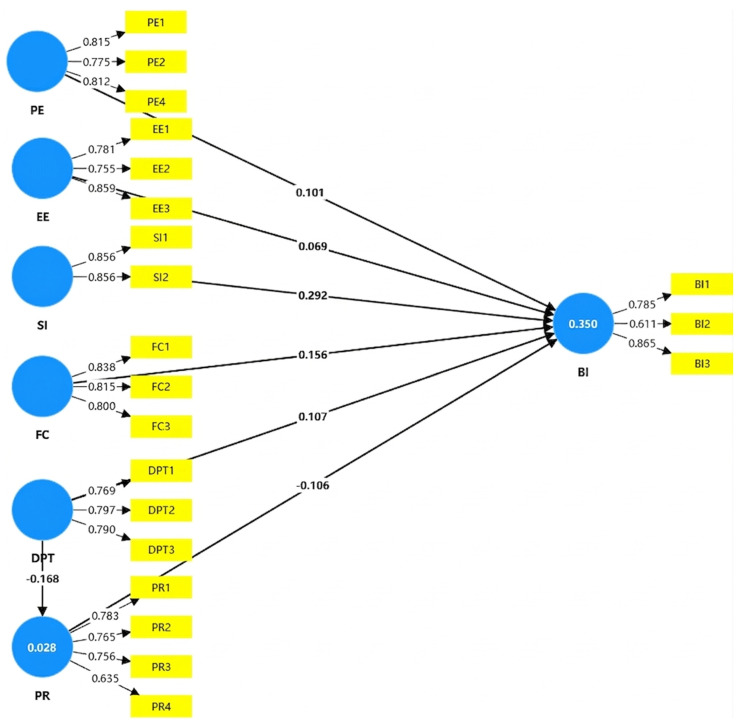
Values of the path coefficients and outer loadings after the PLS-SEM algorithm run. Blue circles represent latent constructs; yellow boxes denote observed indicators. Values on arrows from constructs to indicators are outer loadings; values on arrows between constructs are standardized path coefficients. The R^2^ value is shown in the endogenous construct circle. PE = Performance Expectancy; EE = Effort Expectancy; SI = Social Influence; FC = Facilitating Condition; DPT = Doctor–Patient Trust; PR = Perceived Risk; BI = Behavioral Intention. Statistical significance was evaluated at conventional thresholds (*p* < 0.05).

The results of the structural equation model are shown in Figure 2, and the PLS results of the hypotheses tests are presented in Table 6. Table 6 summarizes the path coefficients and hypothesis testing results of the SEM. The predictive power of the model was verified through the bootstrapping algorithm. The results show that the relationships between Interpersonal Social Influence (i.e., family and peer endorsement) and BI (t = 8.748, β = 0.292, *p* < 0.001), FC and BI (t = 4.185, β = 0.156, *p* < 0.001), PE and BI (t = 2.850, β = 0.101, *p* = 0.004), and DPT and BI (t = 3.167, β = 0.107, *p* = 0.002) were significant. Additionally, PR and BI (t = 3.730, β = −0.106, *p* < 0.001) and DPT and PR (t = 4.686, β = −0.168, *p* < 0.001) also exhibited significant path coefficients. Furthermore, the mediation analysis reveals that PR partially mediates the relationship between DPT and BI. Both the direct effect (β = 0.107, *p* = 0.002) and indirect effect (β = 0.018, *p* = 0.003) are statistically significant, indicating that DPT influences BI both directly and through the reduction of PR. Thus, H1, H3, H4, H5, H6, H7 and H8 were statistically supported. However, the association between EE and BI (t = 1.806, β = 0.069, *p* = 0.071) was not statistically significant. Thus, in this study, H2 was not supported at the conventional significance level.

As shown in Figure 2, BI exhibited an R^2^ of 0.350, suggesting that the predictors accounted for a moderate level of variance. Despite the statistical significance of the path coefficient (β = −0.168) and mediation effect (β = 0.018), the low R^2^ (0.028) for PR indicates weak explanatory power, suggesting that the impact of this specific pathway is limited. The standardized root mean square residual (SRMR) was used to assess model fit, with values below 0.08 indicating a good fit [66]. The SRMR value for the model was 0.069, indicating an acceptable fit for the data.

### 4.5. Multi-Group Analysis Results

Education-based subgroup comparison: A permutation-based multi-group analysis was conducted to compare respondents with higher education and lower education. As shown in Table 7, the structural relationships between the main constructs were largely consistent across educational groups. None of the path coefficient differences reached statistical significance (all *p* > 0.05). Although some variations in the magnitude of path coefficients were observed—for example, SI showed a stronger association with BI among respondents with higher education (β = 0.313) compared with those with lower education (β = 0.179)—the permutation test indicated that these differences were not statistically significant (*p* = 0.066). These findings suggest that the proposed model operates similarly across different educational levels.

Age-based subgroup comparison: A similar multi-group analysis was conducted comparing respondents aged below 50 years and 50 years or older (Table 8). As reported in Table Y, the path coefficients were generally comparable across age groups. None of the structural path differences between the two age groups were statistically significant (all *p* > 0.05). For instance, SI had a somewhat stronger effect on BI among younger respondents (β = 0.286) compared with older respondents (β = 0.246), but the difference was not statistically significant (*p* = 0.591). Overall, these results indicate that the structural relationships in the proposed model are relatively stable across age groups.

## 5. Discussion

This study provided a comprehensive empirical analysis of the determinants associated with BI to adopt AI-assisted lung cancer screening by extending the UTAUT framework to incorporate DPT and PR. The findings highlighted the significant associations of PE, SI, FCs, and DPT with BI. Additionally, PR was negatively associated with BI. DPT demonstrated a dual association. It was directly associated with BI and indirectly associated with adoption through the mitigation of PR.

### 5.1. Main Findings

The results indicate that Perceived Risk was negatively associated with BI. This suggests that individuals who perceive greater uncertainty or potential negative consequences are less willing to adopt AI-assisted lung cancer screening. This finding is consistent with prior research on health technology adoption [34]. PR has been identified as an important psychological barrier that may discourage individuals from accepting emerging digital health technologies. In the context of AI-assisted medical applications, PR is often multidimensional. Our measurement items reflected concerns about diagnostic reliability and data privacy. However, previous literature has highlighted additional sources of uncertainty. These include algorithmic bias, lack of transparency in AI decision-making, and ambiguity regarding responsibility for adverse outcomes [37,67]. Such concerns may collectively shape individuals’ broader perceptions of technological uncertainty and institutional trust when evaluating AI-assisted health services. Moreover, the integration of AI into medical screening may also introduce perceived systemic risks related to the transformation of traditional physician–patient relationships and the redistribution of decision-making authority between human clinicians and algorithmic systems. Perceived reductions in human oversight may lead to more cautious stances toward AI-assisted screening [64].

Doctor–Patient Trust emerged as a significant factor associated with BI to accept AI-assisted lung cancer screening. The positive direct association suggests that trust in healthcare providers appears central to patients’ willingness to accept emerging medical technologies. In clinical contexts characterized by uncertainty and technical complexity, patients often rely on trusted physicians as heuristic decision anchors. Thus, trust may serve as a relational bridge associated with acceptance of AI-supported diagnostic tools. DPT also demonstrated a dual pathway through partial mediation: it is directly associated with BI and indirectly associated with adoption through the mitigation of PR. One possible interpretation is that, for many residents, AI-based diagnostic systems remain relatively opaque and difficult to evaluate directly. In such contexts, physicians may serve as an important interpretive reference point. Established trust in doctors may extend to their recommended technologies. This trust appears associated with reduced concerns about data privacy, diagnostic errors, and system reliability [39]. As PR decreases, individuals may become more willing to accept AI-assisted screening, consistent with the observed mediation pathway. However, the explanatory power of DPT for PR was modest (R^2^ = 0.028), suggesting that trust is associated with reduced, but does not fundamentally account for, risk perception. Consistent with this pattern, the indirect association of DPT on BI through PR was statistically significant but small in magnitude (β = 0.018). Therefore, the mediation pathway should be interpreted as a supplementary mechanism, whereas the primary influence of DPT operates through its direct effect on BI. This phenomenon reflects the profound complexity of risk perception in oncological screenings. In the context of lung cancer, which is associated with high mortality, a physician’s interpersonal endorsement (DPT) attenuates but does not fully resolve the multifaceted anxieties patients face with AI-assisted screening. The relatively low R^2^ value indicates that a substantial proportion of variance in PR remains unexplained within the current model. This finding underscores the complexity of risk perception in oncological screening contexts. While physician endorsement may buffer uncertainty, PR is likely associated with broader dispositional and contextual factors, such as baseline AI literacy, elevated health anxiety related to lung cancer, institutional trust, and perceptions of systemic accountability. Accordingly, reducing PR may require not only improvements in clinical communication but also broader institutional strategies, including clearer regulatory frameworks and enhanced transparency in AI decision-making processes.

Interpersonal Social Influence (operationalized as the SI construct in this study) emerged as the strongest predictor of BI, emphasizing the central role of close social networks and normative endorsement. This contrasts with consumer-oriented technologies, where decisions often rely on personal judgment. Healthcare decisions, particularly concerning new technologies like AI, show strong associations with external recommendations. This is particularly true in the medical context, characterized by considerable information asymmetry between healthcare providers and patients. Existing literature supports the idea that, in health decisions, subjective norms—such as encouragement from family and peers—appear to serve as important guiding forces [65]. As new technologies like AI-assisted screening are not easily understood by the general public, these social cues are associated with reduced uncertainty and increased adoption.

Facilitating Conditions are significantly associated with BI, underscoring the importance of the structural and resource-related aspects in the adoption of AI-assisted screening. This finding aligns with the UTAUT model’s notion that adoption is determined not only by motivation but also by the availability of supportive resources. In the context of healthcare, factors such as the accessibility of AI-equipped hospitals, the affordability of screening services, and the availability of trained medical personnel are crucial for ensuring that individuals can realistically engage with the technology.

Performance Expectancy is positively associated with BI, suggesting that individuals are more likely to adopt AI-assisted lung cancer screening when they believe it will enhance diagnostic accuracy and improve screening outcomes. Although the effect size of PE was smaller than those of SI and FC, its impact remained statistically significant. This result supports the idea that perceived benefits are a core driver of adoption intention, a finding consistent with prior research on technology acceptance among patient groups [68]. In medical settings, where early diagnosis and accurate results are critical for survival, the perceived utility of AI in improving diagnostic performance is strongly associated with adoption.

Effort Expectancy was not significantly associated with BI in this study. To ensure this finding was not a statistical artifact, we first conducted a rigorous assessment of the EE construct. As shown in Table 4, the EE items were positively evaluated (mean range = 3.93–4.19 on a five-point scale) and exhibited adequate variability (SD range = 0.51–0.70). Importantly, the magnitude of variance observed for EE was comparable to that of the other latent constructs in the model, suggesting that restricted variance or ceiling effects are unlikely to account for the non-significant structural relationship. Given the moderate association observed between EE and FC, we additionally examined whether multicollinearity or conceptual redundancy might explain this finding. However, collinearity diagnostics indicated that variance inflation factors were well below recommended thresholds, and discriminant validity criteria were satisfied. These results suggest that the non-significant effect of EE is unlikely to be attributable to statistical overlap with FC. This pattern may instead reflect the specific clinical context of AI-assisted lung cancer screening, where passive patient participation reduces the cognitive burden on individuals, thereby diminishing the salience of effort-related concerns in adoption decisions. The finding that EE may not be significantly associated with BI in certain healthcare contexts is supported by research showing no association between PE and BI to use AI in some studies [69]. This suggests that the relationship between EE and adoption may be context-dependent. It may be understood through the perspective of “Offloading under Cognitive Load” [70]. Traditional mobile medical apps require users to personally learn interfaces. Thus, usability is strongly associated with adoption. However, AI-assisted lung cancer screening represents a ‘passive medical scenario.’ Patients need only cooperate with routine CT scans. Radiologists undertake the high cognitive burden of operating AI and interpreting reports. For patients, AI technology is ‘invisible.’ They need not understand algorithms. They simply follow doctors’ final advice based on AI results. Therefore, in this specific medical context, the perceived level of effort proved less critical to patients’ decision-making.

The stratified multi-group analysis further indicates that the structural relationships in the proposed model remain largely stable across age and educational subgroups, suggesting that the model may also be applicable to populations that are more representative of the clinical target group for lung cancer screening.

### 5.2. Practical Implications

Based on the statistically significant determinants identified in the structural model, this study also provides several practically relevant implications for the implementation of AI-assisted lung cancer screening programs.

First, the findings highlight the importance of Perceived Risk and Doctor–Patient Trust in shaping public acceptance of AI-assisted screening. The negative association between PR and BI suggests that residents remain concerned about issues such as diagnostic accuracy, data privacy, and medical liability related to AI technologies. From a practical perspective, establishing robust regulatory and legal frameworks is therefore essential. Clear policies regarding data protection, diagnostic accountability, and clinical responsibility should be developed. For example, when radiologists issue AI-assisted screening reports, a manual review signature and professional endorsement could be included to reinforce accountability. Such measures may provide the “psychological safety net” necessary to reduce public concerns and improve confidence in AI-assisted screening systems.

At the same time, doctor–patient trust appears to play a critical role in mediating public acceptance of AI-based medical technologies. In clinical practice, physicians often act as interpreters between complex technologies and patients. When physicians explain AI-generated results, contextualize them within clinical judgment, and transparently communicate both the benefits and limitations of AI systems, patients may be more likely to perceive these technologies as supportive tools rather than opaque “black boxes.” Training healthcare professionals to effectively communicate AI-assisted diagnostic results and integrating AI outputs into routine doctor–patient consultations may therefore strengthen trust and facilitate the adoption of AI-assisted screening services.

Second, the results indicate that interpersonal social influence plays an important role in shaping BI. In the Chinese social context, health decisions are often embedded within family and community networks rather than being purely individual choices. As a result, generic health education campaigns may be insufficient. Instead, targeted community-based mobilization strategies may be more effective. For example, the “Family Doctor Contract Service” could be leveraged, as family doctors serve as trusted public health gatekeepers who maintain long-term relationships with residents. Integrating recommendations for AI-assisted screening into routine follow-up services provided by family doctors may therefore enhance public acceptance. In addition, trusted community figures and peer opinion leaders—such as health volunteers or residents who have benefited from early detection—may help shape positive public perceptions. Community-based health workshops or informal health salons where these individuals share their experiences may help reduce skepticism toward “black-box” technologies and foster more favorable attitudes toward AI-assisted screening.

Finally, improving Facilitating Conditions and Performance Expectancy is essential for translating positive attitudes into actual adoption behavior. Policymakers should ensure that AI-assisted screening services are both accessible and affordable. This may involve expanding the distribution of AI-equipped screening facilities and incorporating screening costs into existing medical insurance or public health subsidy programs. By lowering economic and logistical barriers while clearly communicating the efficiency and diagnostic benefits of AI-assisted screening, policymakers can help ensure that residents’ positive expectations of the technology are supported by adequate system-level resources.

### 5.3. Strength and Limitations

A key strength of this study lies in its focus on the specific context of AI-assisted lung cancer screening. By incorporating DPT and PR into the UTAUT framework, this research extends the model, which may better reflect relational and psychological dimensions that are highly relevant to medical AI adoption, thereby enhancing its contextual applicability and explanatory scope. In addition, the use of a nationwide, stratified sampling strategy provides a broad empirical basis for examining public acceptance of AI-assisted screening across diverse regions and populations in China, which may improve the general relevance of the findings.

This study has several limitations that should be acknowledged.

First, issues related to sample composition and representativeness should be considered. The data-quality screening procedures, while enhancing internal validity, led to the exclusion of approximately one-third of initial responses. These procedures may have disproportionately excluded individuals with lower engagement, limited health literacy, or less survey experience. Consequently, the final sample may overrepresent urban and highly educated populations, which limits the generalizability of findings to these groups. In addition, although we employed a nationwide survey with a multistage stratified sampling strategy covering all 31 provincial-level administrative regions in mainland China, the final sample was still dominated by younger and highly educated respondents. Consequently, the findings may not fully represent older or less educated populations, who constitute the primary target group for lung cancer screening programs. Future research should prioritize recruiting larger and more representative samples of screening-eligible individuals, for example through targeted recruitment in clinical settings (e.g., respiratory clinics or smoking cessation programs), mixed-mode survey designs incorporating telephone or in-person data collection, or oversampling strategies for high-risk demographic strata.

Second, the cross-sectional nature of the data limits the ability to draw causal inferences. Although the estimated structural relationships are consistent with the proposed theoretical framework, they reflect directional associations rather than causal effects. Longitudinal or experimental studies are therefore needed to determine whether the identified determinants, such as trust and PR, translate into actual adoption behaviors over time.

Third, the reliance on self-reported data may introduce response biases, including social desirability bias or potential misinterpretation of survey items. Although several procedural safeguards were implemented—such as interviewer assistance for concept clarification, assurances of anonymity, and data-quality screening procedures—responses may still reflect participants’ subjective perceptions rather than objective behaviors. Future studies could strengthen the robustness of the findings by incorporating behavioral data, longitudinal tracking, or mixed-method research designs.

Fourth, some limitations relate to model specification and construct operationalization. The model demonstrated relatively limited explanatory power for PR, suggesting that risk perceptions regarding AI-assisted screening may also be influenced by additional factors not included in the current framework, such as technological literacy, media exposure, or prior experiences with digital health technologies. Future studies could incorporate these factors to provide a more comprehensive understanding of the formation of risk perceptions toward medical AI. In addition, the operationalization of Social Influence was constrained by measurement considerations. The removal of the media-related item resulted in a two-item construct primarily capturing interpersonal normative influence. Although reliability and validity criteria were satisfied, this reduction narrows the conceptual breadth of the construct. Future research could examine interpersonal endorsement and media influence as separate constructs to provide a more comprehensive understanding of SI mechanisms in AI adoption contexts. In addition, there is potential for semantic overlap in the measurement of some constructs. For instance, one BI item (“…I am more willing to accept the decisions made by doctors taking into consideration the examination results based on this AI technology”) directly references physician, which is conceptually close to the domain of DPT. Although statistical tests supported discriminant validity, this wording may have inflated the observed relationship between the two constructs. Future studies could use more distinct item phrasings to minimize this potential conflation.

Finally, BI was modeled as a proxy for potential adoption of AI-assisted lung cancer screening. Although BI is widely recognized as a strong predictor of technology adoption in frameworks such as UTAUT and the Theory of Planned Behavior, it may not always translate into actual usage behavior in real-world healthcare settings. The BI construct in this study also exhibited modest internal consistency (Cronbach’s alpha = 0.636), although composite reliability exceeded conventional thresholds. Future research could refine item wording, introduce additional items to improve scale reliability, or examine whether different expressions of acceptance—such as personal adoption intention, comparative evaluation, and willingness to recommend—should be modeled as distinct but related outcome constructs. Longitudinal or real-world implementation studies are also needed to examine whether individuals’ intentions ultimately translate into actual screening participation.

## 6. Conclusions

This study suggests that Social Influence, Facilitating Conditions, Performance Expectancy, Doctor–Patient Trust, and Perceived Risk are significantly associated with residents’ BI to adopt AI-assisted lung cancer screening. Furthermore, Doctor–Patient Trust is indirectly associated with BI through Perceived Risk. These findings suggest that in addition to improving infrastructure and external service conditions, strengthening trust relationships and addressing risk perceptions are essential for promoting the effective uptake of AI-assisted screening. Accordingly, AI-assisted lung cancer screening programs may be more effectively implemented by combining targeted interpersonal communication through family doctors and community opinion leaders, clear physician–patient explanations of AI outputs to build trust, and system-level support to enhance accessibility, affordability, and perceived utility.

## Figures and Tables

**Table 1:** Measurement items for the survey questionnaire.

Serial Number	Variable	Measurement Item Content	Adapted from
Determinants			
1	Performance Expectancy (PE)	I believe this AI technology can reduce the occurrence of missed diagnoses or misdiagnoses.	Venkatesh [16] Tian, W [43]
		I believe this AI technology can shorten the waiting time for screening reports.	
		I believe this AI technology can provide me with personalized health advice (such as specific precautions, follow-up intervals, etc.).	
2	Effort Expectancy (EE)	Providing clear and understandable guidelines will increase my willingness to accept it.	Venkatesh [16] Calisto [31] Md Masud Rana [44] Yao [39]
		Not requiring additional preparation for this AI technology beyond what is required for regular CT scans will increase my willingness to accept it.	
		Being able to conveniently view the report of this AI technology through digital devices such as mobile phones will increase my willingness to accept it.	
3	Social Influence (SI)	My family supports my use of this AI technology.	Venkatesh [16] Md Masud Rana [44] Lanying Wang [45]
		My friends or colleagues support my use of this AI technology.	
		Positive coverage of this AI technology on social media will increase my willingness to accept it.	
4	Facilitating Conditions (FCs)	Providing a priority queue for booking appointments for this AI-assisted lung cancer screening will increase my willingness to accept it.	Venkatesh [16] Ryo Fujimori [46]
		Reimbursement by basic medical insurance or other government healthcare subsidy programs for the cost of this AI technology will increase my willingness to accept it.	
		Being able to undergo this AI-assisted lung cancer screening at polyclinics will increase my willingness to accept it.	
5	Doctor–Patient Trust (DPT)	I believe doctors are of the required professional level to judge the accuracy or validity of the results of this AI technology.	Tiantian Ye [38]
		I believe doctors will explain the AI results or listen to my experience and suggestions about this service.	
		Doctor’s recommendation of this AI technology will increase my willingness to accept it.	
6	Perceived Risk (PR)	I believe there are no risks with this AI technology worth worrying about.	Featherman [34]
		I am worried that the results of this AI technology may be inaccurate, posing potential medical risks.	
		I am worried that when this AI technology makes a wrong diagnosis, the medical responsibility may not be clear.	
		I am worried that my health information in this AI technology may be leaked without authorization.	
Response Variable			
7	Behavioral Intention (BI)	Within the next six months, I am willing to proactively undergo an AI-assisted lung cancer screening.	Cheng [27] Zhang [29]
		Overall, compared to the traditional radiographic examination without AI, I am more willing to accept the decisions made by doctors taking into consideration the examination results based on this AI technology.	
		I will recommend others to use this AI technology.	

**Table 2:** Characteristics of participants and extended UTAUT constructs (n = 971).

	Total (N = 971)	High BI (n = 504)	Low BI (n = 467)	*p*-Value
Sex				0.129
Male	489 (50.4%)	242 (48%)	247 (52.9%)	
Female	482 (49.6%)	262 (52%)	220 (47.1%)	
Age (years)				0.064
18–35	465 (47.9%)	229 (45.5%)	236 (50.5%)	
36–55	420 (43.3%)	221 (43.8%)	199 (42.6%)	
>55	86 (8.9%)	54 (10.7%)	32 (6.9%)	
Education Level				0.601
Junior high school or below	104 (10.7%)	56 (11.1%)	48 (10.3%)	
High school/Associate degree	516 (53.1%)	260 (51.6%)	256 (54.8%)	
Bachelor’s degree and above	351 (36.1%)	188 (37.3%)	163 (34.9%)	
Monthly Household Income (CNY)				0.313
Low (<5000)	298 (30.7%)	154 (30.6%)	144 (30.8%)	
Medium (5000–15,000)	639 (65.8%)	328 (65.1%)	311 (66.6%)	
High (>15,000)	34 (3.5%)	22 (4.3%)	12 (2.6%)	
Residence				0.069
Urban area	853 (87.8%)	452 (89.7%)	401 (85.9%)	
Rural area (town/village)	118 (12.2%)	52 (10.3%)	66 (14.1%)	
Heard of Medical AI				**<0.001**
Yes	597 (61.5%)	345 (68.5%)	252 (54.1%)	
No	374 (38.5%)	159 (31.5%)	214 (45.9%)	
Extended UTAUT constructs, median (Q1, Q3)				
Performance Expectancy (PE)	4.00 [3.67, 4.33]	4.00 [4.00, 4.33]	4.00 [3.67, 4.00]	**<0.001**
Effort Expectancy (EE)	4.00 [3.33, 4.00]	4.00 [3.67, 4.33]	3.67 [3.33, 4.00]	**<0.001**
Social Influence (SI)	3.33 [3.00, 3.67]	3.33 [2.67, 3.67]	3.33 [3.00, 4.00]	**<0.001**
Facilitating Conditions (FCs)	4.00 [3.67, 4.33]	4.00 [4.00, 4.33]	4.00 [3.67, 4.00]	**<0.001**
Doctor–Patient Trust (DPT)	4.00 [4.00, 4.33]	4.00 [4.00, 4.67]	4.00 [3.67, 4.33]	**<0.001**
Perceived Risk (PR)	4.00 [4.00, 4.67]	4.33 [4.00, 4.67]	4.00 [3.67, 4.33]	**<0.001**

Note: CNY, Chinese Yuan. Participants were categorized into High BI (coded as one) and Low BI (coded as two) groups based on their total BI score, using a cut-off value of four. Bold text indicates that group comparison between high BI and low BI was statistically significant at *p* < 0.05 based on Bartlett’s test and a chi-squared test.

**Table 3:** Results of exploratory factor analysis.

Items	Factor Loading Coefficient							Communality
	Factor 1	Factor 2	Factor 3	Factor 4	Factor 5	Factor 6	Factor 7	
PR1	0.767							0.592
PR2	0.835							0.715
PR3	0.848							0.738
PR4	0.791							0.634
FC1		0.711						0.631
FC2		0.756						0.694
FC3		0.702						0.595
PE1			0.776					0.687
PE2			0.728					0.649
PE3			0.755					0.587
DPT1				0.778				0.669
DPT2				0.771				0.664
DPT3				0.624				0.537
EE1					0.765			0.566
EE2					0.758			0.654
EE3					0.709			0.642
SI1						0.788		0.75
SI2						0.830		0.77
SI3		0.327	0.268			0.452		0.515
BI1							0.711	0.637
BI2							0.723	0.695
BI3							0.760	0.631
Eigenvalue	2.33	2.229	2.051	2.014	1.972	1.819	1.699	-
Variance explained ratio	10.59%	10.13%	9.32%	9.15%	8.96%	8.27%	7.72%	-
Cumulative variance explained ratio	10.59%	20.73%	30.05%	39.20%	48.16%	56.43%	64.155%	-
KMO measure	0.897							
Bartlett’s sphericity test	Approx. chi-square: 6776.044							
Df	231							
*p*-value	0.000							

**Table 4:** Structural reliability and convergent validity.

Construct	Mean	SD	Outer Loading	Cronbach’s Alpha	Composite Reliability (rho_a)	Composite Reliability (rho_c)	Average Variance Extracted (AVE)
PE	3.94–4.14	0.55–0.65	0.775–0.815	0.721	0.727	0.843	0.641
SI	3.69–3.71	0.57–0.72	0.856	0.783	0.784	0.902	0.822
PR	3.02–3.47	0.81–0.92	0.635–0.783	0.75	0.822	0.825	0.543
DPT	3.92–4.04	0.55–0.60	0.769–0.797	0.691	0.712	0.829	0.617
EE	3.93–4.19	0.51–0.70	0.755–0.859	0.719	0.741	0.841	0.639
FC	4.08–4.36	0.57–0.64	0.800–0.838	0.753	0.757	0.858	0.669
BI	3.71–3.92	0.63–0.72	0.611–0.865	0.636	0.701	0.802	0.58

**Table 5:** Discriminant validity.

Construct	PE	SI	PR	DPT	EE	FC	BI
	Heterotrait–monotrait ratio (HTMT)						
PE							
SI	0.590						
PR	0.273	0.307					
DPT	0.582	0.462	0.201				
EE	0.558	0.443	0.141	0.671			
FC	0.6	0.437	0.143	0.598	0.791		
BI	0.604	0.640	0.282	0.576	0.569	0.607	
	Fornell–Larcker criterion						
PE	**0.801**						
SI	0.446	**0.907**					
PR	−0.223	−0.254	**0.737**				
DPT	0.412	0.341	−0.168	**0.786**			
EE	0.476	0.335	−0.119	0.477	**0.8**		
FC	0.443	0.337	−0.12	0.436	0.59	**0.818**	
BI	0.419	0.475	−0.242	0.387	0.39	0.422	**0.761**

Note: Bolded diagonal elements are the square root of the AVE for each construct.

**Table 6:** Path coefficients by bootstrapping.

Hypothesis	Path	Std. Β (β)	STD	t-Value	*p*-Value	Result
H1	PE → BI	0.101	0.035	2.85	0.004	Supported
H2	EE → BI	0.069	0.038	1.806	0.071	Not supported
H3	SI → BI	0.292	0.033	8.748	<0.001	Supported
H4	FC → BI	0.156	0.037	4.185	<0.001	Supported
H5	DPT → BI	0.107	0.034	3.167	0.002	Supported
H6	PR → BI	−0.106	0.028	3.73	<0.001	Supported
H7	DPT → PR	−0.168	0.036	4.686	<0.001	Supported
The mediation effect test						
H8	DPT → PR→ BI	0.018	0.006	2.953	0.006	Supported

**Table 7:** Multi-group analysis by educational level (permutation test).

Path	Higher Education Group (β)	Lower Education Group (β)	Difference (Δβ)	Permutation 2.5%	Permutation 97.5%	*p*-Value
PE → BI	0.105	0.174	−0.068	−0.150	0.154	0.388
EE → BI	0.077	0.073	0.004	−0.166	0.166	0.958
SI → BI	0.313	0.179	0.134	−0.140	0.143	0.066
FC → BI	0.156	0.236	−0.080	−0.164	0.165	0.350
DPT → BI	0.096	0.180	−0.083	−0.155	0.149	0.278
PR → BI	−0.101	−0.035	−0.066	−0.120	0.128	0.298
DPT → PR	−0.180	−0.183	0.003	−0.155	0.161	0.977
DPT → PR → BI	0.018	0.006	0.012	−0.030	0.022	0.375

Note: β = standardized path coefficient. Δβ represents the difference between subgroup coefficients. Statistical significance of group differences was assessed using the permutation test in PLS-SEM. None of the path differences reached statistical significance at the 0.05 level.

**Table 8:** Multi-group analysis by age group (permutation test).

Path	Age < 50 Group (β)	Age ≥ 50 Group (β)	Difference (Δβ)	Permutation 2.5%	Permutation 97.5%	*p*-Value
PE → BI	0.128	0.081	0.048	−0.155	0.161	0.562
EE → BI	0.059	0.157	−0.099	−0.173	0.162	0.249
SI → BI	0.286	0.246	0.040	−0.143	0.146	0.591
FC → BI	0.175	0.190	−0.016	−0.172	0.169	0.852
DPT → BI	0.093	0.180	−0.087	−0.158	0.152	0.278
PR → BI	−0.086	−0.070	−0.016	−0.127	0.134	0.807
DPT → PR	−0.188	−0.143	−0.045	−0.160	0.165	0.601
DPT → PR → BI	0.016	0.010	0.006	−0.032	0.023	0.661

Note: β = standardized path coefficient. Δβ represents the difference between subgroup coefficients. Statistical significance of group differences was assessed using the permutation test in PLS-SEM. None of the path differences reached statistical significance at the 0.05 level.

## Data Availability

The data presented in this study are available on reasonable request from the corresponding author due to the ongoing nature of the research project, which involves confidentiality and ethical considerations regarding unpublished findings.

## Abbreviations

The following abbreviations are used in this manuscript:

UTUAT	Unified Theory of Acceptance and Use of Technology
AI	Artificial Intelligence
EFA	Exploratory Factor Analysis
PLS-SEM	Partial Least Squares Structural Equation Modeling
BI	Behavioral Intention
CT	Computed Tomography
TRA	Theory of Reasoned Action
TPB	Theory of Planned Behavior
TAM	Technology Acceptance Model
PE	Performance Expectancy
EE	Effort Expectancy
SI	Social Influence
FC	Facilitating Condition
DPT	Doctor–Patient Trust
PR	Perceived Risk
S-CVI	Content Validity Index of the Scale
I-CVI	Item Content Validity Index
CATI	Computer-Assisted Telephone Interviewing
KMO	Kaiser–Meyer–Olkin
PCA	Principal Component Analysis
AVE	Average Variance Extracted
CR	Composite Reliability
STD	Standard Deviations
HTMT	Heterotrait–Monotrait ratio
VIF	Variance Inflation Factor
SRMR	Standardized Root Mean Square Residual
